# Fast Calcium Imaging with Optical Sectioning via HiLo Microscopy

**DOI:** 10.1371/journal.pone.0143681

**Published:** 2015-12-01

**Authors:** Marcel A. Lauterbach, Emiliano Ronzitti, Jenna R. Sternberg, Claire Wyart, Valentina Emiliani

**Affiliations:** 1 Wavefront-Engineering Microscopy Group, Neurophotonics Laboratory, CNRS UMR8250, University Paris Descartes, Sorbonne Paris Cité, Paris, France; 2 Institut du Cerveau et de la Moelle Epinière (ICM), INSERM U1127, CNRS UMR 7225, Sorbonne Paris Cité, University Pierre et Marie Curie Paris 06 UMR S 1127, Paris, France; Institut Curie, FRANCE

## Abstract

Imaging intracellular calcium concentration via reporters that change their fluorescence properties upon binding of calcium, referred to as calcium imaging, has revolutionized our way to probe neuronal activity non-invasively. To reach neurons densely located deep in the tissue, optical sectioning at high rate of acquisition is necessary but difficult to achieve in a cost effective manner. Here we implement an accessible solution relying on HiLo microscopy to provide robust optical sectioning with a high frame rate *in vivo*. We show that large calcium signals can be recorded from dense neuronal populations at high acquisition rates. We quantify the optical sectioning capabilities and demonstrate the benefits of HiLo microscopy compared to wide-field microscopy for calcium imaging and 3D reconstruction. We apply HiLo microscopy to functional calcium imaging at 100 frames per second deep in biological tissues. This approach enables us to discriminate neuronal activity of motor neurons from different depths in the spinal cord of zebrafish embryos. We observe distinct time courses of calcium signals in somata and axons. We show that our method enables to remove large fluctuations of the background fluorescence. All together our setup can be implemented to provide efficient optical sectioning *in vivo* at low cost on a wide range of existing microscopes.

## Introduction

Calcium imaging enables the study of activity in large populations of neurons with high sensitivity and high frame rates [1]. Camera-based imaging in wide-field (WF) mode can be fast, but imaging in thick specimen might require optical sectioning to suppress spurious out-of-focus signals [2]. Since the brain is an inherently three-dimensional structure, such sectioning is essential for resolving neuronal signals and deciphering its function. Two-photon excitation microscopy achieves optical sectioning at high penetration depth in highly scattering media; accordingly, it is the common approach for deep calcium imaging studies in the brain. On the contrary, for thin samples or weakly-scattering tissue-investigations, single-photon (1P) excitation can be sufficient and optical sectioning is usually provided by confocal laser scanning microscopy [3]. The major limitation of a 1P scanning system with respect to a WF mode is essentially the acquisition speed. Implementations with resonant scan mirrors or acousto-optical deflectors [4] can increase scan speed but they remain point scanning techniques. Apart from technical constraints, the available fluorescence signal limits ultimately the dwell time and hence the achievable speed.

Methods enabling WF imaging, where all pixels of the image are recorded in parallel, while preserving the optical sectioning capability of the point scanning methods would be optimal. Optical approaches have been conceived based either on a parallelization of the confocal principle using multiple pinholes (Spinning Disk Confocal Microscopy, SDCM) [4], on a rearrangement of the illumination to induce excitation only in the focal plane (Single/Selective Plane Illumination Microscopy, SPIM) [5, 6], or on a spatial modulation of the light to encode only in-focus information (Structured Illumination Microscopy, SIM) [7–9]. Although SDCM can image at video rate, inter-pinhole cross-talk impairs the out-of-focus light rejection in thick tissue with dense labeling [10]. SPIM demands side-on access and imposes constraints on handling of the sample, limiting its applications.

SIM offers a compromise between recording speed, sectioning capability, ease of integration into a standard WF microscope, and cost. Optical sectioning by SIM is essentially based on the elementary property of a WF microscope to blur fine details of the image when moving the sample away from the focal plane [11]. SIM structures the excitation light in order to create artificially fine high-contrasted details in the sample. This enables recognizing the in-focus portion of an object (the in-focus portion is the one where details are seen sharply), even in the absence of intrinsic details of the sample. A few raw images are computationally combined to separate the artificially superimposed structures in the image from intrinsic details and to calculate an optically sectioned image, i.e. to isolate the part of the object in focus. Relatively high imaging speed is maintained as the total frame rate is equivalent to the one in WF mode just reduced by a factor corresponding to the number of raw images required for the computation of the sectioned image.

HiLo microscopy [8] is a SIM technique, which needs only two images: one image with structured illumination and one with conventional uniform illumination (“HIgh” and “LOw” contrast images). Structured illumination either with a grating or with laser speckles introduces fine details that enable the computation of the optically sectioned image. Combination with the uniformly illuminated image allows recovering all structural information without the need to combine several non-uniform illumination patterns [12]. HiLo imaging can be fast, since only two raw images are needed to obtain the sectioned image. It has been used for imaging *c*. *elegans* and *danio rerio* (zebrafish) at 7 frames per second (fps) [13], chick embryos with up to 9.5 fps [14], *xenopus laevis* embryos with 25 fps [15] and externally induced calcium signals at cellular resolution in the brain of *drosophila melanogaster* with 30 fps [16].

Here, we present a fast optical configuration for HiLo microscopy that enables rapid switching between structured and uniform illumination to obtain optically sectioned images, thus allowing high-speed image recording (up to 100 fps) and demonstrate its capabilities by recording calcium transients in motor neurons in the embryonic *danio rerio* spinal cord.

## Results

**Fig 1:**
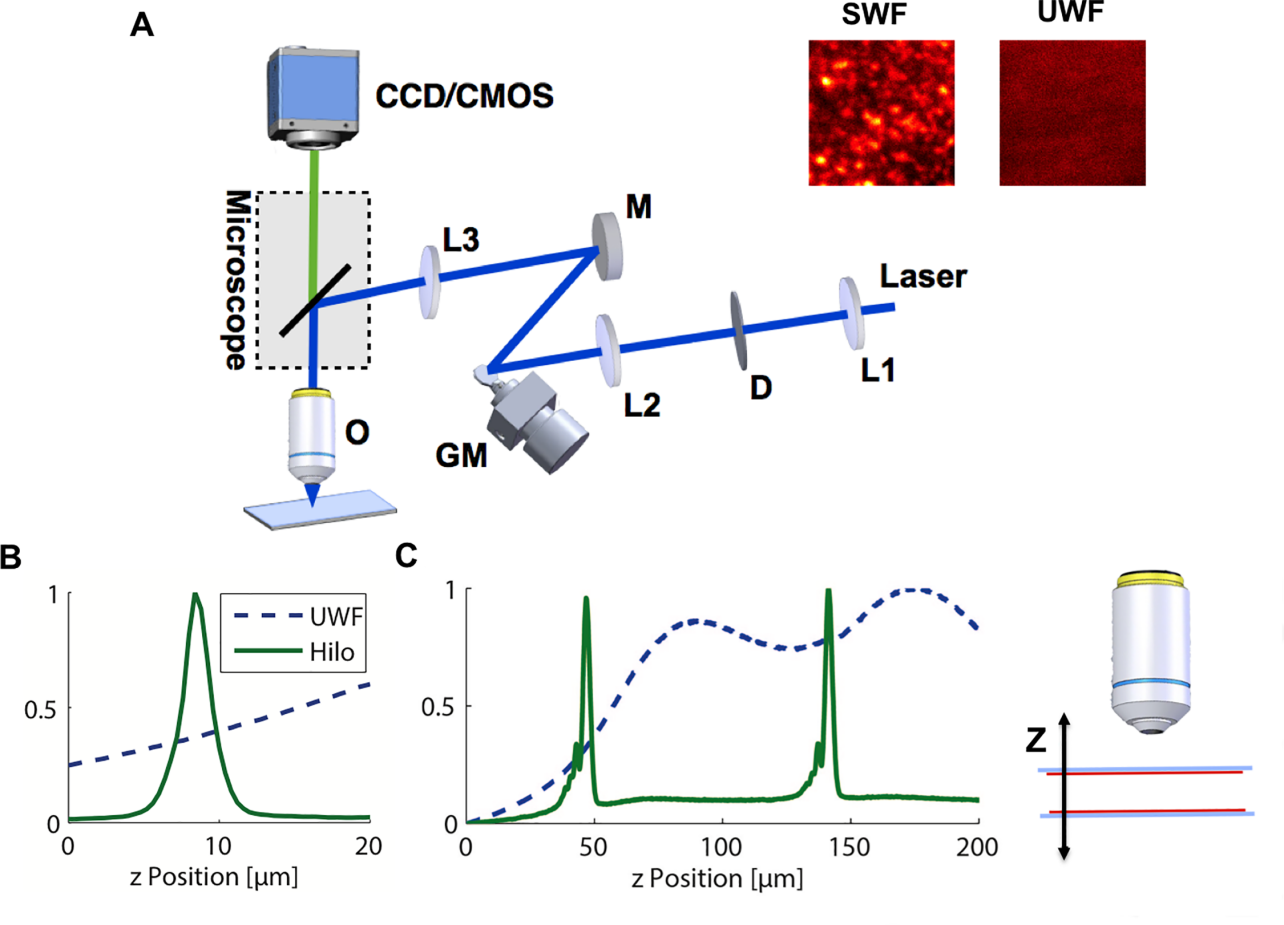
Implementation of HiLo Microscopy to enable optical sectioning at high frame rate in thick biological tissue. (**A**) Setup: A 473nm laser beam is expanded with lens L1 to a divergent beam to illuminate the diffuser D. The diffuser is imaged by lens L2 onto the galvanometric mirror GM. The galvanometric mirror is then imaged by lens L3 into a conjugated plane of the back focal plane of the objective O within the microscope body. Fluorescence images are acquired either with a CCD camera or a CMOS camera. (**Inset**) Fluorescent Rhodamine layer imaged with speckled (SWF) and uniform (UWF) widefield illumination at 200 frames per second (fps), confirming successful uniform/speckled illumination. (**B–C**) Illustration of the optical Sectioning: (**B**) A single fluorescent layer appears as a sharp intensity peak (width 2.1 μm) in a z-stack in HiLo mode (solid green line), but with almost constant intensity in uniform wide-field (UWF) mode (blue dotted line). (**C**) Two Rhodamine layers can be separated in HiLo mode (solid green line), but in UWF mode only broad peaks (due to spherical aberration) whose positions do not correspond to the layers are visible. Inset: Experimental arrangement: red: Rhodamine layers; gray: supporting coverslips.

The optical set up for HiLo microscopy is built up around a commercial upright microscope (Fig 1). In order to realize high-frame-rate HiLo, fast switching between the speckled (SWF) and the uniform widefield illumination (UWF) is required. This is here achieved with a static diffuser imaged onto a galvanometric mirror, which is in turn projected into the back focal plane of the objective (Fig 1A). Each position of the galvanometric mirror generates a differently shifted speckle pattern (Fig 1A inset, SWF) at the sample plane. When the mirror is vibrated with a period that is much shorter than the exposure time of the imaging camera, the different speckle patterns are averaged giving a uniform illumination (Fig 1A inset, UWF). With this configuration we could acquire HiLo images up to 100 fps.

To quantify the optical sectioning achievable in our HiLo microscope, we recorded a z-stack of a thin fluorescent layer and determined the fluorescence intensity (averaged over the whole image) as a function of Z position (Fig 1B). Whereas UWF microscopy showed an almost constant intensity with no sectioning (Fig 1B, dashed blue line), HiLo microscopy showed a sharp peak at the position of the fluorescent layer (Fig 1B, green line; full width at half maximum (FWHM) = 2.1 μm).

To achieve efficient optical sectioning *in vivo*, the ability to separate several objects along the optical axis is critical. We tested this with two fluorescent layers spaced by 94 μm (Fig 1C). UWF microscopy showed broad intensity peaks due to spherical aberrations that do not correspond to the position of the fluorescent layers (Fig 1C, dashed blue line). On the contrary, HiLo microscopy permits to clearly separate the two fluorescent layers showing sharp peaks at 47 μm and 141 μm Z position (Fig 1C, green line).

**Fig 2:**
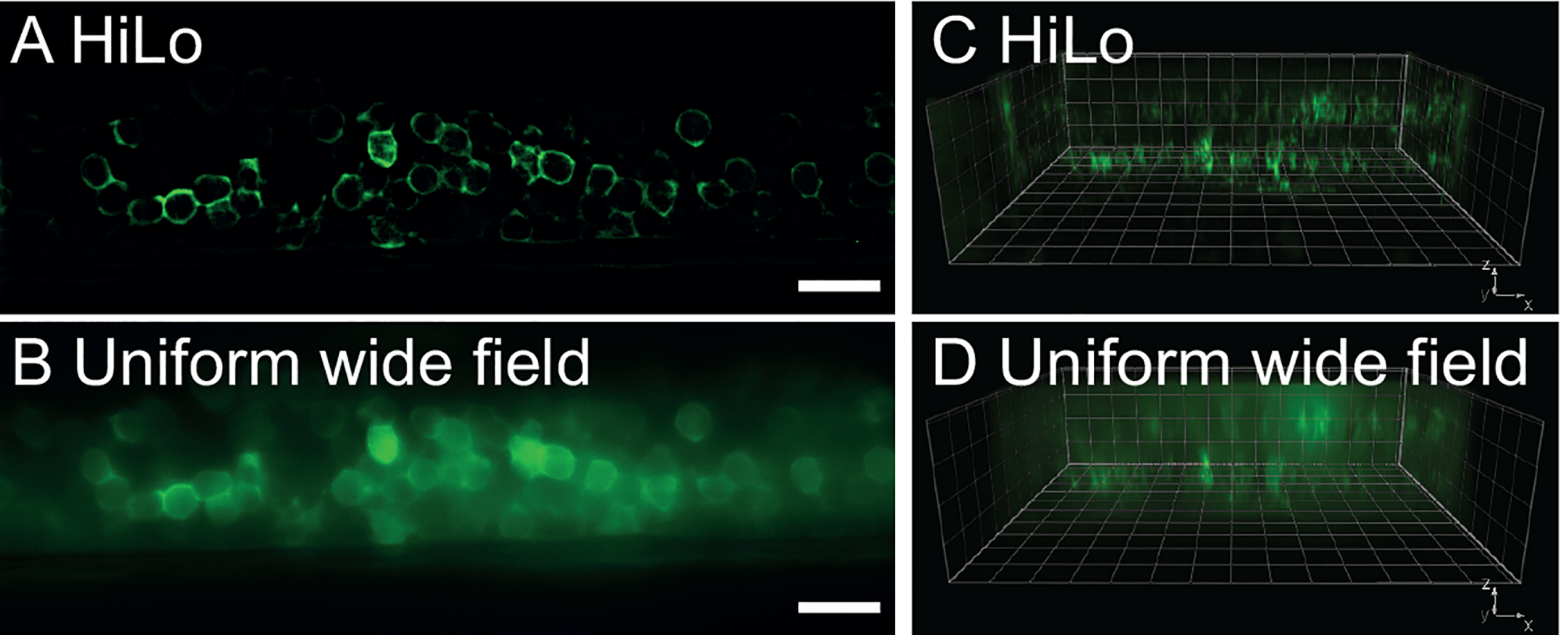
Optical section in embryonic spinal cord of zebrafish obtained with HiLo microscopy. (**A**) Lateral view of the spinal cord of transgenic zebrafish embryo expressing the fluorescent protein GCaMP5 pan-neuronally in *Tg(elavl3*:*GCaMP5G)* obtained with HiLo microscopy. **(B)** Same image obtained in UWF mode. The spatial distribution of GCaMP5G remains hidden under a haze of background fluorescence due to missing optical sectioning.Scale bars in both panels are 20 μm. **(C)** Corresponding z-stack in 3D visualization, imaged in HiLo mode. The pools of neurons on each side of the spinal cord are visible in two separate planes. **(D)** Same reconstruction in WF mode (grid step 10 μm/line). See also S1 Movie.

In order to demonstrate the capability of the microscope for optical sectioning in living samples, we imaged the nervous system of zebrafish embryos (between the 26- and the 30-somite-stage) from transgenic animals expressing genetically encoded calcium indicator GCaMP5 [17] in either motor neurons with *Tg(UAS*:*GCaMP5G* ^*icm08*^ *; Et(e1b*:*Gal4-VP16)s1020t)* or pan-neuronally with *Tg(elavl3*:*GCaMP5G)* [18–20]. The HiLo microscope enabled the visualization of optical sections revealing the distribution of GCaMP5 (Fig 2A), not visible in epifluorescence illumination (Fig 2B). Finally, we performed a three-dimensional reconstruction of the neurons in the spinal cord of the embryo imaged from a lateral view (Fig 2C and 2D). Neurons from both sides of the embryonic spinal cord were clearly visualized due to the sectioning capability of the HiLo microscopy (Fig 2C, S1 Movie) while they remained blurred with UWF illumination (Fig 2D).

The high-frame-rate acquisition reachable with our implementation of HiLo microscopy was then used to record calcium transients in zebrafish embryos expressing GCaMP5 at the embryonic 26- to 30-somite stage, when motor neurons are alternatively activated on the left and on the right side of the embryo [21, 22].

**Fig 3:**
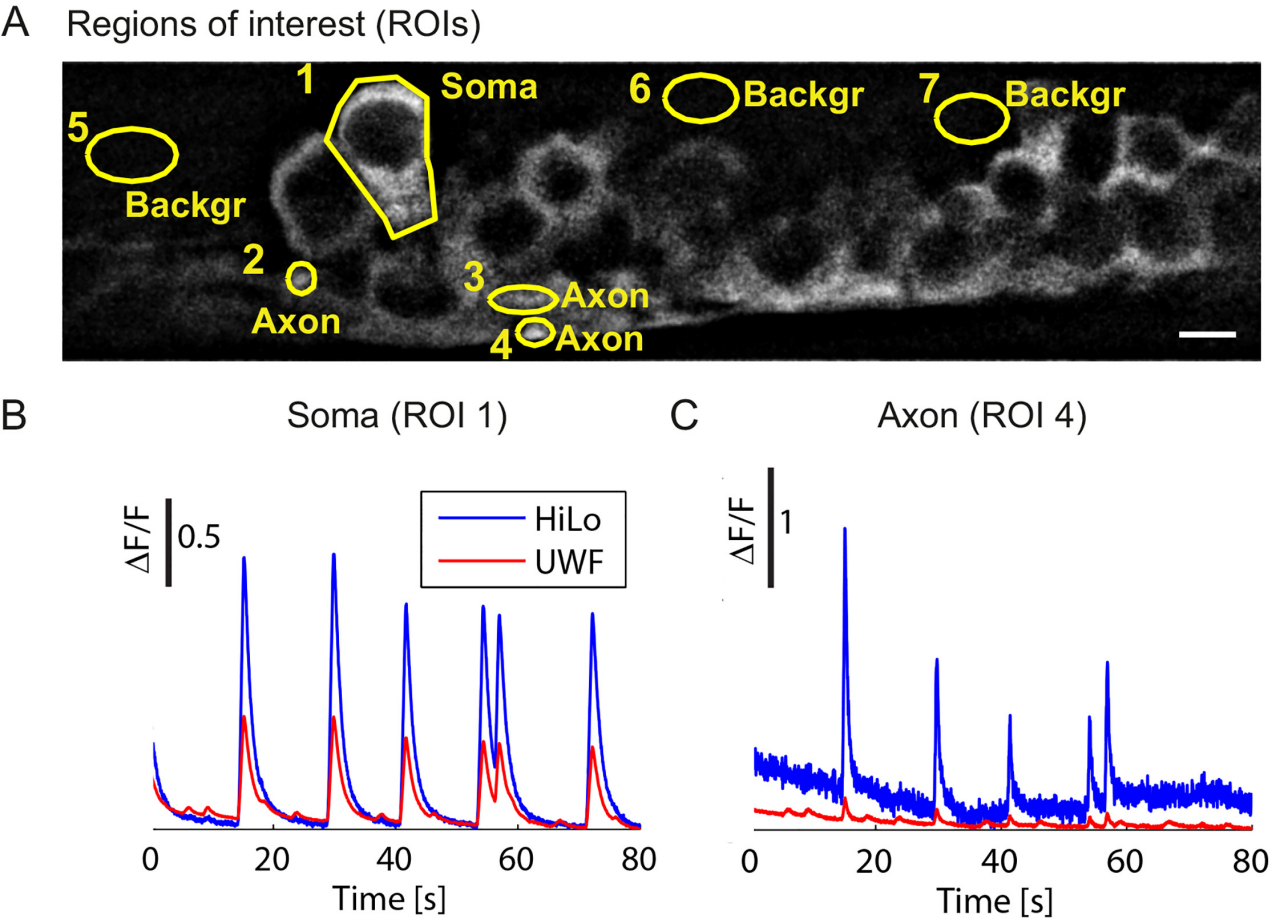
HiLo microscopy enhances calcium signals. (**A**) *In vivo* HiLo image of motor neurons of a zebrafish embryo expressing GCaMP5G in soma (ROI1), axons (ROIs 2–4), or background (ROIs 5–7). Scale bar 5 μm. (**B**) ΔF/F time series in the soma (ROI 1) shows that HiLo mode leads to a larger signal than UWF mode. (**C**) For axons (ROI 4) the gain in HiLo mode is even larger. Acquisition rate was 25fps.

In order to study the HiLo capability for calcium imaging, fluorescence variations (ΔF/F) of the calcium indicator measured from selected regions of interest (ROIs; cell bodies, axons and background) in HiLo and UWF mode have been compared in *Tg(Et(e1b*:*Gal4-VP16)s1020t; UAS*:*GCaMP5G* ^*icm08*^ *)* (Fig 3A). Two main benefits can be noted in the HiLo mode:

First, substantially increased ΔF/F amplitudes are observable with HiLo (Fig 3, S2 Movie). Already on the cell bodies, which gave a strong signal, HiLo microscopy led to a gain of a factor of 2.5 in signal-to-baseline ratio (ΔF/F) compared to UWF microscopy. (Fig 3B) This gain reached up to a factor 10 for less bright regions as axons (Fig 3C). Such an activity detection boost, due to a reduction of the out-of-focus stray light contribution, reveals the higher sensitivity of HiLo for identifying cellular activity with respect to a conventional WF observation.

**Fig 4:**
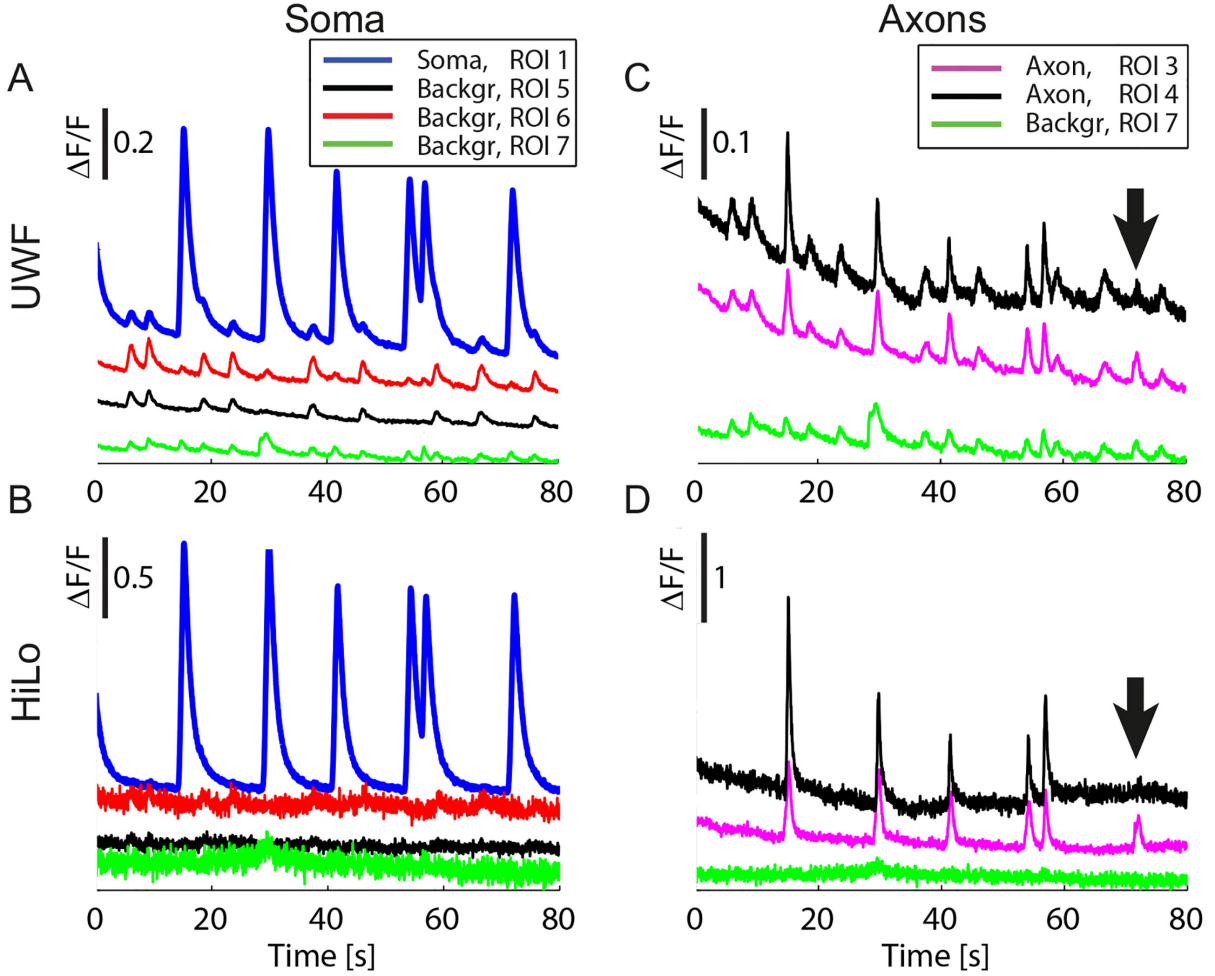
Suppression of non-specific signals. Nonspecific signals appeared in UWF mode (A, C) and were suppressed in HiLo mode (B, D) (ROIs numbered as in Fig 3A). The benefit is consistent in the somatic signal (A, B) and particularly important for the axonal signals (C, D) which are smaller and therefore more difficult to distinguish from the unspecific signals. Furthermore HiLo mode allows observing that calcium spikes occasionally did not occur in a single axon (black trace, arrow), despite occurrence in the neighboring axon (magenta trace) (D), while UWF mode hindered the observation of this phenomenon (C). Note that some bleaching is seen over the 80 s recording time. Traces were offset vertically for visualization. Raw data are shown in all panels. Acquisition rate was 25 fps.

Second, a set of lower-level peaks present in the UWF calcium signals (Fig 4A)was suppressed in the HiLo recording (Fig 4B). Peculiarly, contrary to the principal peaks, these secondary peaks were not localized to a specific ROI and were present also in the background signal (Fig 4A, Background ROIs 5–7). These secondary fluorescence variations are thus not linked to an activity of the targeted cell, but originate from out-of-focus planes where anti-correlated neuronal activity was produced from the pool of contralateral motor neurons [21, 22]. Although for relatively strong signals, the real specific targeted activity can be distinguished from the out-of-focus contributions on the basis of the amplitude (Fig 4A), in many other circumstances the two contributions become hardly distinguishable, possibly leading to an erroneous interpretation of the cellular activity (Fig 4C and 4D). Being able to acquire only the fluorescence signal from the in-focus region of the sample, HiLo suppresses such unwanted peaks both in the cells and in the background (Fig 4B and 4D). Especially, we were able to resolve with HiLo microscopy that neuronal activity is not always transmitted to all cells: ROI 4 on the axonal track does not show any calcium signal at 72 s (Fig 4D, black trace, arrow), despite a strong signal in the adjacent ROI (ROI 3, magenta trace in Fig 4D). This missing peak is in stark contrast with all other calcium peaks in this recording, which were faithfully spread to all ROIs (peaks at 15 s, 30 s, 42 s, 54 s, 57 s). Normal wide-field imaging shows a spurious signal at this time point (Fig 4C, black trace, arrow), which is indistinguishable from true neuronal signals. Only HiLo microsopy allowed here to detect the singular absence of signal transmission.

Thus, compared to conventional widefield microscopy, HiLo microscopy offers a higher sensitivity for fluorescence-variation detection and higher specificity in terms of spatial localization of the calcium signals. Since a fundamental requirement of calcium-signal recording is being able to follow fast cellular kinetics, such HiLo benefits can become of authentic advantage if HiLo imaging can be performed at very high frame rates.

**Fig 5:**
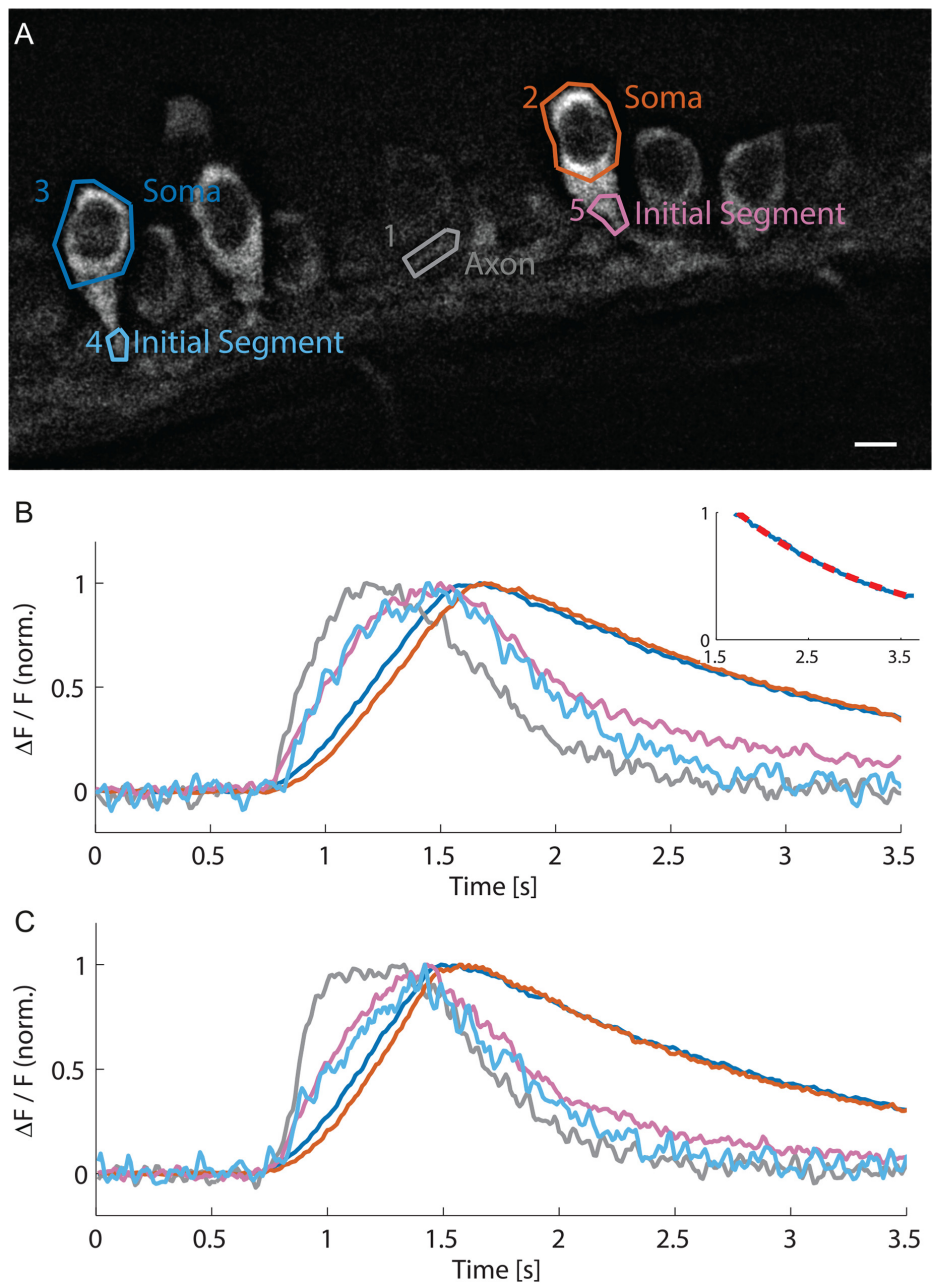
Fast calcium imaging at 100 fps with HiLo microscopy reveals different dynamics in soma and initial segment. (**A**) HiLo image of motor neurons expressing GCaMP5G with ROIs. Scale bar 5 μm. (**B**) Time course of calcium signals in soma, initial segment and axon, recorded at 100 fps. In the axon (ROI 1, gray) and the initial segments (ROI 4, light blue; ROI 5, magenta) a much faster rise is observed than in the somata (ROI 2, orange; ROI 3, dark blue). Somatic signals show a linear rise and a mono-exponential decay (Inset: blue somatic signal with red dotted fit, decay time 1.7 s, R^2^ = 0.998). (**C**) The kinetics of calcium transients in different compartments were stable over time as shown with a recording performed 2.5 min later.

We therefore verified HiLo-calcium-imaging performance up to 100 fps (200 fps raw-data rate) (Fig 5, S3 Movie), a frame-rate at which cellular sub-compartmental kinetics can be extensively investigated. This temporal resolution allows clearly observing that calcium transients rose first in axons and then in the soma (Fig 5A and 5B). Moreover, the intracellular calcium concentration rise was faster in the initial segment (Fig 5A and 5B, ROI 4, ROI 5, light blue and magenta) than in the rest of the soma (ROI 2, ROI 3, orange and dark blue). In the soma a close to linear rise of intracellular calcium was observed, followed by an exponential decay with a time constant of 1.7 s (Fig 5C, Inset: blue data/red-dotted fit). The sequence and kinetics of the calcium transients were stable over time, as seen when recording neuronal activity 2.5 min later (Fig 5C).

## Discussion

We have demonstrated high rate *in vivo* HiLo microscopy on zebrafish embryos enabling fast calcium imaging up to 100 fps on a field of view of ~100 μm x 50 μm with optical sectioning.

We have shown an axial resolution of ~2 μm, permitting to resolve single axonal and dendritic processes. Correct targeting of the genetically encoded marker GCaMP5G could be visualized without the haze of out-of-focus fluorescence due to subcellular resolution.

The optical sectioning provided by HiLo microscopy increased the amplitudes of the recorded calcium signal and permitted the rejection of out-of-focus signals originating from motor neurons in the contralateral side. This suppression of spurious calcium signals by HiLo microscopy allowed a distinction between small signals that originate from the structure of interest and stray signals from other neurons. This capability is particularly relevant for a correct interpretation and localization of the neuronal signals. Indeed in UWF, the stray signals originating from neuronal activity from out-of-focus planes can be almost as strong as the signal arising from small regions of observation as axonal portions, making a distinction of true signals and spurious signals almost impossible (Fig 4).

HiLo recordings have been shown with up to 100 fps, demonstrating that fast cellular dynamics can be studied. This high imaging rate allows resolving differences in rise time of calcium transients for soma and axon. The calcium-concentration rise in the initial segments was faster than in the soma. By eliminating the out-of-focus light, we could detect failures to generate spikes in some axons.

It is worth mentioning that speckles are an inevitable interference phenomenon in laser illumination [23]. Fully developed speckles are therefore resistant against misalignment and optical aberrations [24]. Compared to alternative HiLo methods that project gratings into the sample plane, speckle projection is therefore more robust to optical aberrations induced by the microscope and sample.

We demonstrate that a fast-switching design can allow following calcium signals in zebrafish up to 100 fps, permitting accurate analysis of calcium kinetics. The system has an extremely simple design using a standard static diffuser in combination with a one-axis scan mirror that has to operate just in two states: stationary and moving. This is achieved by turning a sinusoidal command voltage on and off.

In this study, we demonstrated single-color HiLo imaging; however, extension to multiple colors would be straightforward and only require adding a second laser and the corresponding filter cube without further changes in the optical path. In addition HiLo microscopy can easily be combined with electrophysiology. Also combination with other optical widefield methods remains possible, e.g. to stimulate neurons optogenetically either via uniform [25] or targeted [26] illumination. Application to functional imaging other than calcium concentration, e.g. imaging of voltage-sensitive dyes should be feasible as well.

High-frame-rate optical sectioning for dynamic imaging can be alternatively obtained via Spinning Disk Confocal Microscopy (SDCM) or Single Plane Illumination Microscopy (SPIM). SDCM extends the confocal principle with a parallelized detection through multiple pinholes to speed up the scanning. Although a single-pinhole physical rejection of out-of focus-light is often superior over computational SIMs in thick tissues [9], with multi-pinhole detection the inter-pinhole cross-talk hampers the optical sectioning, leading to background signals [21,22]. Thus, when optical sectioning and high-frame rate are required as for calcium imaging, HiLo is an advantageous alternative to SDCM. Moreover, confocal microscopy requires lasers of high beam quality as this determines the reachable resolution, while for HiLo microscopy the demand on laser-beam quality is lower, because the laser illuminates just a diffuser.

SPIM delivers sidewise illumination to illuminate just a single plane of the sample. Since the optical sectioning is fully provided through a selective plane illumination, the detection can be fully equivalent to a conventional microscope, thus basically speed-limited only by the camera performance and available signal [18, 27]. However, the need for two orthogonal optical paths restricts the access to the sample. representing a major limitation. Moreover, the sample scattering and aberrations can broaden and distort the plane of illumination, reducing the optical sectioning. On the contrary, clear access to the sample is maintained in HiLo since it is based on WF epifluorescence imaging.

## Conclusions

In summary this study highlights a simple, easy-to-implement and cost-efficient method for fast calcium imaging in thick low-scattering samples. Optical sectioning is achieved due to alignment- and aberration-insensitive structured illumination with laser speckles. Images are acquired on a camera as in conventional WF microscopy, which is fast since no scanning is necessary. We showed here that this approach was powerful to perform *in vivo* calcium imaging with a genetically encoded calcium sensor at a speed of 100 fps on motor neurons of the zebrafish embryonic spinal cord.

## Materials and Methods

### Optical setup

A HiLo microscope using laser speckles for structured illumination (Fig 1A) was implemented on a commercial microscope body (Axio Examiner.Z1, Zeiss). A laser (MBL-F-473-500, Laser 2000, Wessling, Germany) with an emission wavelength of 473 nm and a power of 500 mW served as light source. The beam was expanded to a divergent beam with lens L1 (Fig 1A) to illuminate a static diffuser D (scattering angle 1°, 20DKIT-C1, Newport, Irvine, CA, USA). The diffuser was then imaged onto the galvanometric mirror GM (Cambridge Technology) with lens L2. Since the mirror had a size of only 4 mm, this image was demagnified. Note that a 4f geometry that preserves collimation of beams is not necessary. The galvanometric mirror was then imaged with lens L3 (f = 30 mm) through the rear port of the microscope onto an intermediate back focal plane in the microscope stand. The illumination was reflected to the objective O (Zeiss W Plan-Apochromat 63x/1.0) with a filter cube (Semrock, FITC-3540C-000) of the microscope. Fluorescence images were acquired either with a CCD camera (Coolsnap ES^2^, Photometrics, Tucson, AZ, USA,) or a CMOS camera (Orca Flash, C11440, Hamamatsu, Massy, France). Illumination power was 2.8 mW.

The microscope and the CCD camera were controlled with the software Slidebook (Intelligent Imaging Innovations, Denver, CO, USA), the CMOS camera with the software HCImage (Hamamatsu).

To obtain structured illumination, the galvanometric mirror was kept stationary; for uniform illumination it was vibrated with a frequency much faster than the frame rate. To this end the galvanometric mirror was vibrated with a sine wave (700 Hz) from a function generator (203047, Jeulin, Evreux, France).

The vibration could be turned on and off via a TTL signal, which allowed control of the vibration via the microscope’s software Slidebook. Alternatively this TTL signal was derived directly from the camera. The frame clock of the camera was divided by two with custom built electronics, giving a high TTL signal (galvo moving) for every other frame.

### Fluorescent layers

Fluorescent layers for validation of optical sectioning (Fig 1B and 1C) were made of Rhodamine 6G in polymethyl-methacrylate (PMMA): 20 μl of a Rhodamine 6G solution (1M Rhodamine in a 2% w/v PMMA-in-chloroform solution) were spin coated onto glass cover slips (#1, 25 mm, BK-7, diameter 18 mm, Marienfeld Superior, Menzel-Gläser GmbH, Braunschweig, Germany) at 8500 rpm.

The fluorescent double layer for testing the optical sectioning (Fig 1B and 1C) was fabricated by gluing two such spin coated cover slips together with double-sided adhesive tape. The Rhodamine layers were facing the tape. The space between the cover slips was filled with water.

### Zebrafish care and strains

*Danio rerio* embryos of the AB and Tubingen Longfin (TL) genetic backgrounds were raised in egg water [28], at 28.5°C until 75% epiboly and then at 26°C to delay development for experimentation until the 24-somite stage. GCaMP5G-positive embryos originating from the cross of *Tg(Et(e1b*:*Gal4-VP16)s1020t; UAS*:*GCaMP5G* ^*icm08*^ *)* or *Tg(elavl3*:*GCaMP5G)* with wild type adults were dechorionated, mounted laterally in 1.5% agar at the 24-somite stage, and paralyzed with injections of 0.5 mM α-bungarotoxin (Sigma-Aldrich, USA) into caudal muscle of the tail. Experiments were performed at the 26–30 somite stages at room temperature. Embryo staging was performed according to stages described in Kimmel et al. [29]. All procedures were approved by the Institutional Ethics Committee “Charles Darwin” at the Institut du Cerveau et de la Moelle épinière (ICM), Paris, France, and received subsequent approval from the EEC (2010/63/EU).

### Data analysis

Images were acquired in 16 bit and exported in tiff format. All further data processing was performed with MATLAB (Mathworks, Natick, MA, USA).

#### HiLo

Raw images were recorded alternatingly with uniform illumination and speckled illumination. Optically sectioned images (“HiLo images”) were calculated from pairs of a speckled image (SWF) followed by a uniformly illuminated image (UWF) with the algorithm previously described [12]. A plugin for Image J (Rasband, W.S., ImageJ, U. S. National Institutes of Health, Bethesda, Maryland, USA, http://imagej.nih.gov/ij/, 1997–2014) is available from the group of Jerome Mertz (http://biomicroscopy.bu.edu/). We generated custom made Matlab scripts based on initial code kindly provided by Daryl Lim and Jerome Mertz. We favored the Matlab implementation for greater control of parameters and for the possibility of automating data processing. Since two raw images are used to obtain one HiLo image, the frame rate of the raw images is twice the frame rate of the HiLo images. All UWF images presented in the article are the images with uniform illumination that were also used for the calculation of the HiLo images.

#### Optical sectioning

To test the resolution of optical sectioning (Fig 1), image stacks of fluorescent Rhodamine 6G layers were recorded with a long distance 63X, NA 1.0 water-dipping objective. Fluorescence intensity was averaged over the whole field of view and plotted against axial position. Raw data without further smoothing are displayed.

#### Calcium imaging

For time series of calcium transients, the average fluorescence intensity was determined in the HiLo and the UWF images in ROIs as indicated. Fluorescence changes ΔF were normalized to the minimal fluorescence F in each time series. To show small peaks without overlay with other graphs, traces in Fig 4A and 4C were offset vertically by 0.1, traces in Fig 4B by 0.2, and traces in Fig 4D by 0.4. No temporal averaging was applied to the calcium signals.

## Supporting Information

S1 Fig Same data as in Fig 3C not normalized to ΔF/F, but shown as raw signals. (PDF)

S1 Movie 3D-view of the spinal cord of double transgenic zebrafish embryo expressing the fluorescent protein GCaMP5 in motor neurons obtained with HiLo microscopy. (WMV)

S2 Movie Comparison of calcium transients imaged in HiLo and wide-field modes at 25 fps. (**Upper Panel**) *In vivo* HiLo image of motor neurons of a zebrafish embryo expressing GCaMP5G. Optical sectioning in HiLo mode visualizes the membraneous distribution of GCaMP5G and the calcium transients of spontaneous activity. (**Lower Panel**) Same recording in normal widefield mode with uniform illumination (UWF). Due to a lack of sectioning the localization of GCaMP5G and the calcium transients remain hidden in a haze.
(MP4)

S3 Movie Stop-motion imaging of calcium transients at 100 fps. HiLo microscopy reaches up to 100 fps (upper panel) and provides the necessary optical sectioning, whereas the neurons are not clearly resolved in widefield mode (UWF) (lower panel).
(MP4)
