# Supplementary figures and images for: Fast Calcium Imaging with Optical Sectioning via HiLo Microscopy
Source: PLoS One. 2015 Dec 1;10(12):e0143681. doi: 10.1371/journal.pone.0143681 (PMC4666667; doi:10.1371/journal.pone.0143681)

# Axon (ROI 4)

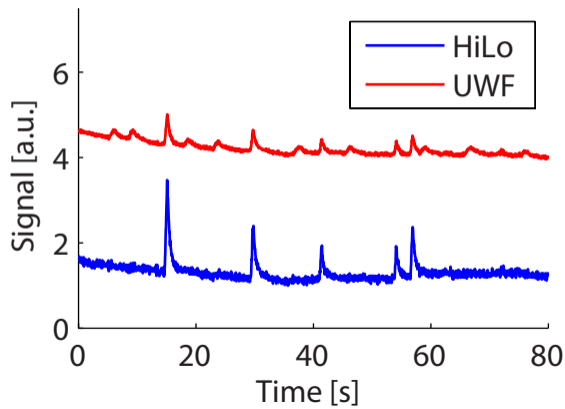

Supplement: S1 Fig — (PDF) [file pone.0143681.s001.pdf]
